# Variables Controlling Entry into and Exit from the Steady-State, One of Two Modes of Feeding in *Aplysia*

**DOI:** 10.1371/journal.pone.0045241

**Published:** 2012-09-28

**Authors:** Nimrod Miller, Silvia Marcovich, Abraham J. Susswein

**Affiliations:** Leslie and Susan Gonda (Goldschmied) Multidisciplinary Brain Research Center, Mina and Everard Goodman Faculty of Life Sciences, Bar Ilan University, Ramat Gan, Israel; Freie Universitaet Berlin, Germany

## Abstract

**Background:**

*Aplysia* feeding is a model system for examining the neural mechanisms by which changes in motivational state control behavior. When food is intermittently present, *Aplysia* eat large meals controlled by a balance between food stimuli exciting feeding and gut stimuli inhibiting feeding. However, when food is continuously present animals are in a state in which feeding is relatively inhibited and animals eat little. We examined which stimuli provided by food and feeding initiate steady-state inhibition of feeding, and which stimuli maintain the inhibition.

**Results:**

Multiple stimuli were found to control entry into the steady-state inhibition, and its maintenance. The major variable governing entry into the steady-state is fill of the gut with bulk provided by food, but this stimulus cannot alone cause entry into the steady-state. Food odor and nutritional stimuli such as increased hemolymph glucose and L-arginine concentrations also contribute to inhibition of feeding leading to entry into the steady-state. Although food odor can alone cause some inhibition of feeding, it does not amplify the effect of gut fill. By contrast, neither increased hemolymph glucose nor L-arginine alone inhibits feeding in hungry animals, but both amplify the inhibitory effects of food odor, and increased glucose also amplifies the effect of gut fill. The major variable maintaining the steady-state is the continued presence of food odor, which can alone maintain the steady-state for 48–72 hrs. Neither increased glucose nor L-arginine can alone preserve the steady-state, although they partially preserve it. Glucose and arginine partially extend the effect of food odor after 72 hrs.

**Conclusions:**

These findings show that control of *Aplysia* feeding is more complex than was previously thought, in that multiple inhibitory factors interact in its control.

## Introduction

Feeding in the marine gastropod mollusc *Aplysia* has been used as a model system for examining neural mechanisms underlying changes in state [1]–[5]. In all animals, eating a meal generally causes a change in behavioral state, leading to a decrease in behaviors that reflect a drive to eat [1]. A number of studies indicated that meals in *Aplysia* are controlled by a balance between two stimuli: 1) food stimuli that produce arousal of feeding [6, 7] and then initiate appetitive [8], [9] and consummatory feeding behaviors [7], [10]; 2) stretch of the gut by ingested food counter the excitatory effects of food and inhibit feeding [11]–[13]. Food and gut stretch respectively produce graded excitation and inhibition of feeding [14]. Neurons controlling feeding whose activity is modulated by food stimuli that initiate feeding [2], [15] and by gut stimuli that inhibit feeding [3] have been examined, providing insight into how motivationally relevant stimuli modulate behavior [16].

The balance between excitatory and inhibitory inputs causes *Aplysia* to consume large quantities of food intermittently, in discrete meals. Thus, when hungry *Aplysia* are hand-fed small pieces of food, they satiate only after filling the capacious crop with approximately 15% of their body weight, in the course of a 2–3 hour meal [10]. Twenty-four hours later, when *Aplysia* are again hand-fed a meal, they consume approximately 50% of the volume eaten by hungry animals [10]. The quantity of food eaten, and many parameters of feeding behavior during the meals, can be accounted for by the inhibitory effects of bulk stimuli which distend the crop, which work against the excitatory stimuli provided by food. Food can remain within the crop for over 24 hr [12], providing mechanical stimuli that contribute to satiation, and that quantitatively account for the meal size and meal parameters when food is re-encountered. Sustained exposure to food leads to sensory adaptation or habituation and decreased responsiveness [17]–[19], emphasizing that the effects of gut fill are balanced by increases and decreases in the salience of the food [10].

The hypothesis that gut fill and the salience of food are the primary determinant of meals is consistent with studies that examined the possible regulation of feeding by nutritive stimuli such as glucose. Although glucose concentration in the hemolymph is actively regulated, it has not been shown to affect feeding behavior [20].

Observations of groups of *Aplysia* in the field or in laboratory aquaria are consistent with control of feeding by the slow fill of the gut with large quantities of food. *Aplysia* are grazing animals that often live in close proximity to their food, seaweeds [21, 22]. Like other grazing animals [23–25], *Aplysia* can spend much of the day eating a large quantity of food that is slowly digested. Under certain conditions, approximately 20% of the time budget is spent feeding [22]. Feeding in the field is often patterned into meals that can extend for a number of hours [21, 26].

More recent studies [27] have shown that the control of *Aplysia* feeding is considerably more complex than was previously thought. The pattern in which large meals are governed by food salience and anterior gut fill is seen only when food is intermittently available, such as when *Aplysia* are provided with a single daily meal (the feeding regimen used in earlier laboratory studies). However, when isolated *Aplysia* are allowed constant access to food they eat relatively little for as long as the food remains present. In this condition there are short, intermittent feeding bouts that *in toto* occupy approximately 40 min of a full day [27]. This pattern of feeding, termed steady-state inhibition, is maintained over many days of constant food access [27]. The quantity of food eaten and patterns of feeding in this condition are not explained by the quantity of food that fills the crop, combined with the salience of the food [27]. The variables controlling feeding in isolated *Aplysia* with steady-state food access have not been explored. This report examines the variables controlling feeding when *Aplysia* have constant food access, with the aim of later determining how these variables affect identified neurons that control *Aplysia* feeding [27].

When an isolated, hungry *Aplysia* is first provided with *ad libitum* access to food, it eats an initial large meal, and then enters the steady state in which it eats little [27]. Thus, stimuli associated with the initial large meal initiate the steady-state in which feeding is relatively inhibited. One aim of the present study was to identify the stimuli provided by the initial meal that cause the change in state. When animals are in the steady-state and eat relatively little, in spite of constant food access, removing the food causes a gradual decline in the steady-state inhibition. By 24 hours after the removal of the food animals are again hungry, and access to food again initiates a large meal [27]. Thus, stimuli provided by the presence of food and by occasional feeding maintain the steady-state inhibition. A second aim of the study was to determine the stimuli that maintain the steady-state inhibition.

Our data show that multiple stimuli affect both entry into and exit from the steady-state. The major variable governing entry into the steady-state is fill of the gut with bulk provided by food, but this stimulus is not alone capable of causing entry into the steady-state. Food odor and nutritional stimuli such as increased glucose and L-arginine also contribute to inhibition of feeding leading to entry into the steady-state. The major variable maintaining the steady-state is the continued presence of food odor. However, additional stimuli, such as glucose and L-arginine, also contribute. Thus, *Aplysia* feeding is controlled by multiple inhibitory factors, as is feeding in higher animals.

## Materials and Methods

### Animals

Experiments were performed on *Aplysia californica* weighing 80–120 g purchased from Marinus Scientific (Garden Grove, CA, USA) and Santa Barbara Marine Bio (Santa Barbara, CA, USA). The animals were stored in 600 liter tanks of aerated, filtered Mediterranean seawater maintained at 17°C. Lighting was L:D 12∶12. The food used in all experiments was the seaweed *Ulva lactuca* that was gathered at various sites along the Mediterranean coast of Israel and then stored frozen. Food was thawed before use in an experiment. While in the storage tanks, animals were fed 2–3 times weekly.

### Experimental Conditions

Animals were transferred from the storage tanks to 5 or 10 l aerated experimental aquaria 24 hours prior to an experiment. The aquaria were kept at 23°C. Animals were generally kept one to a 5 l aquarium. Animals were food-deprived for 3 days prior to the experiment.

### Non-nutritive Gel

In some animals some of the post-ingestive consequences a meal were mimicked by injecting into the gut 2.5 ml of non-nutritive bulk, in accordance with procedures described previously [12, 27–29]. The material used in these experiments was beads of poly-acrylamide (Bio-Gel P-60, medium, 130±40 µm (wet), Bio-Rad Laboratories, Richmond, CA, USA) that were hydrated by placing them in an excess of seawater for 3 to 12 hours prior to use. The bulk was injected via a 10 ml syringe that was attached to a tube (approximately 2 mm in diameter) that the animals were induced to swallow by first touching the lips with food, and then placing the tube into the mouth when it opened in response to the food. The tube was gently pushed into the gut, while palpating the animal to determine the location of the tip of the tube. When the tip was felt to be close to the back of the crop the contents of the syringe were injected.

### Food Odor and Food Taste

Animals were placed in a 10 l aquarium in which a partition separated the animal from food on the other side of the partition. To test feeding, food was then added to both sides of the partition.

In a second experiment, the lips were stimulated with food every 5 minutes, and food was maintained in contact with the lips until the animal responded with a bite. The food was withdrawn at the bite, preventing the animals from ingesting the food.

### *Ad Libitum* Feeding after L-arginine or after Glucose Treatment

In one series of experiment, animals were food deprived for 3 days prior to the experiment, and then given *ad libitum* access to food (the seaweed *Ulva*) for 4 hours. Animals were treated with various concentrations of L-arginine, with glucose, or with artificial seawater (ASW), 1 or 2 hours before allowing them access to the food. In some experiments, food was also placed in the aquarium, behind a partition preventing the animals from touching the food, two hours before allowing the animals access to the food. After animals were allowed access to the food, the time spent feeding was measured as in previous experiments [27]: animals were sampled every 5 min, and feeding was noted. In two experiments the quantity of food placed in the water was weighed before and after the 4 hour feeding, as previously described [30]. Food was weighed before and after it was put into the aquaria containing an animal, and the difference in the weight was used as a measure of the quantity eaten. Experimental and control animals were matched for weight.

## Results

### Entry into the Steady-state

**Figure 1:**
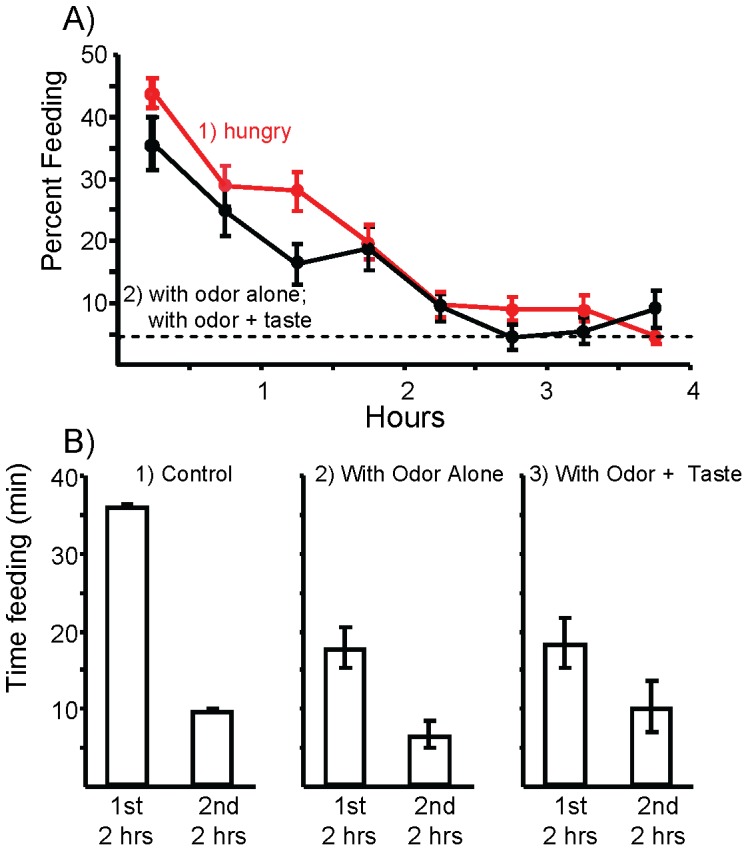
The initial meal in hungry animals, and in animals pre-exposed to the odor and taste of food. A) 1: During a 4 hr exposure to food, the percent time spent feeding gradually decreased. Based on data from previous studies, animals entered the steady-state (dashed line) (*N* = 60 animals). 2) Combined data from experiments measuring feeding after a 2 hour pre-exposure to food odor alone or to the odor plus the taste of food. *N* = 21 animals pre-exposed to food odor; *N* = 16 animals pre-exposed to food odor + taste. B) A comparison of the total time spent feeding during the first and second halves of the 4 hr test in: 1: Hungry animals, and in 2: animals pre-exposed for 2 hr to food odor or 3: to food odor plus the taste of food. Pre-exposure to food odor significantly reduced feeding during the first 2 hr, with respect to feeding during the first 2 hr in hungry animals (for odor: *p*<0.001; *t*(79) = 4.87). However, feeding was significantly higher than that during the second two hours of the test in previously hungry animals (*p* = 0.002, *t*(79) = 2.83), indicating that 2 hours of exposure to food odor did not completely substitute for the first two hours of the meal. Addition of taste to the odor stimulus caused no significant changes in either the first (*p* = 90, *t*(35) = 0.14) or the second (*p* = 0.3, *t*(35) = 1.04; all comparisons are two-tailed t-tests with Bonferroni corrections) halves of the meal. Standard errors are shown.

When abundant food is introduced to hungry animals, *Aplysia* eat a large meal that initiates the steady-state. This is illustrated in Fig. 1A1, which shows the percent time spent feeding per half hour interval when previously hungry animals are allowed a 4 hr access to food. Animals began by devoting 43.9% of their time to feeding during the first 30 min, and ended by devoting 4.7% of the time to feeding during the last 30 min. The level of feeding at the end of the meal is consistent with entry into the steady-state, since previous studies [27] showed that when animals have continuous access to food, they devote 0.5–4% of their time to feeding.

Further analysis of the data showed that animals spent a mean of 46 min feeding during the 4 hrs of food access. This was overwhelmingly during the first 2 hr of food access (78.6% or 36.2 min, versus 21.4% or 9.8 min feeding during the second 2 hr) (Fig. 1B1).

Our initial aim was to examine the stimuli provided by food that contributed to entry into the steady-state, during which feeding is relatively inhibited. Food provides odorants that are sensed by the rhinophores, and tastants that are sensed when food touches the lips [1, 31]. After food is eaten, post-ingestion stimuli could be provided by the bulk of food in the gut and by chemical stimuli in the gut, as well as by nutrients in the hemolymph and in cells, whose concentration changes when food is digested. Any of these factors could be directly sensed by the nervous system, or could induce changes in hormones that are then sensed by the nervous system. We examined the separate effects of various post-ingestion stimuli on the induction of steady state food inhibition.

### Odor and Taste

We examined the possibility that food odor alone may cause entry into the steady-state. The possibility that food odor inhibits feeding seems surprising, in light of the documented ability of food odor to initiate food-finding [32, 33] and biting [1, 34], indicating that food odor excites feeding, rather than inhibiting it. However, a previous study [27] showed that after animals are already in the steady-state, exposure to the odor of food for 24 hr maintains the steady-state inhibition, indicating that food odor can also inhibit feeding.

To test the possibility that exposure to the odor of food could be a stimulus initiating steady-state inhibition, hungry animals were pre-exposed to food odor (provided by food behind a barrier, which could be smelled but which could not be touched) for two hours, and were then allowed 4 hr access to food. If food odor alone has a role in initiating the steady-state inhibition, pre-exposure to odor for 2 hr should reduce the time spent feeding in the subsequent 2 hr. If food odor alone entirely accounts for the effect of food in inducing the steady-state inhibition, the time spent feeding in the first 2 hours that animals can eat should be comparable to the time spent feeding in the second two hours of control animals, when they have already been exposed to food and food odor for two hours.

The pre-exposure to food odor significantly reduced the time spent feeding during the first two hours that food was available by 49.4%, indicating that food odor contributes to the inhibitory effect of food. However, after a 2 hr pre-exposure to food odor significantly more time was spent feeding than after 2 hr of access to food, indicating that food odor cannot alone account for entry into the steady-state inhibition (Fig. 1B2). Additional stimuli provided by the initial meal contributed to the entry into the steady-state.

We examined the possible additional contribution of tasting the food to initiation of the steady state inhibition. For 2 hr prior to the start of the meal, food was touched to the lips every 5 min, eliciting a single attempt to bite before the food was removed. There was no significant difference between the time spent feeding during the first 2 hours of the meal between animals exposed to food odor alone and to food odor plus the taste of the food (Fig. 1B3), indicating tasting the food does not add to entry into the steady state over the effect of food odor alone.

**Figure 2:**
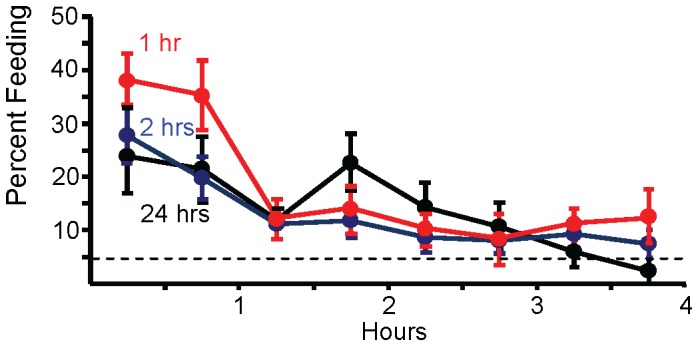
Habituation of excitatory effects of food odor reveals the inhibitory effect. Animals were pre-exposed to food odor for 24 (*N* = 13), 2 (*N* = 27) or 1 (*N* = 18) hours prior to being given access to food. The percent feeding was then measured over the next 4 hours. During the first hour, there was no significant difference in feeding between animals fed after a 24 hr exposure to seaweed odor and animals fed after a 2 hr pre-exposure to the odor (*p* = 0.54, *t*(38) = 0.61; two-tailed *t*-test)). By contrast, there was a significant difference in feeding between animals pre-exposed to food odor for 1 hour and the combined data from 2 and 24 hour pre-exposures to seaweed odor (*p* = 0.01, *t*(56 = 2.60; two-tailed *t*-test).

Since food odor has both excitatory and inhibitory effects on feeding, it is possible that the two hour exposure to food produced a mixture of excitation and inhibition. There is evidence that the excitatory effects of food odor gradually decay due to habituation [18], whereas the inhibitory effects do not habituate over 24 hrs [27], suggesting that a longer exposure to food odor would produce more inhibition of feeding. We tested the possibility that a 24 hr exposure of hungry animals to food odor produces a more profound inhibition of feeding than does a 2 hrs exposure. However, there were no significant differences between animals pre-exposed to food for 2 hrs or for 24 hrs, indicating that a 2 hr exposure to food odor is likely to produce the maximum inhibition that food odor can produce in hungry animals (Fig. 2).

Are shorter pre-exposures to food also effective in maximally inhibiting feeding? By contrast to the effect of a 2 hour pre-exposure to food odor, a 1 hour pre-exposure produced significantly less inhibition than did 2 and 24 hour pre-exposures during the first hour of feeding (Fig. 2). At this time, values were comparable to those in animals that had not been pre-exposed to food odor. This finding indicates that the excitatory effects of food odor decrease over the first 2 hours of exposure to the stimulus, thereby exposing the inhibitory effects, which are maximal by 2 hours after exposure to the food odor.

### Bulk

**Figure 3:**
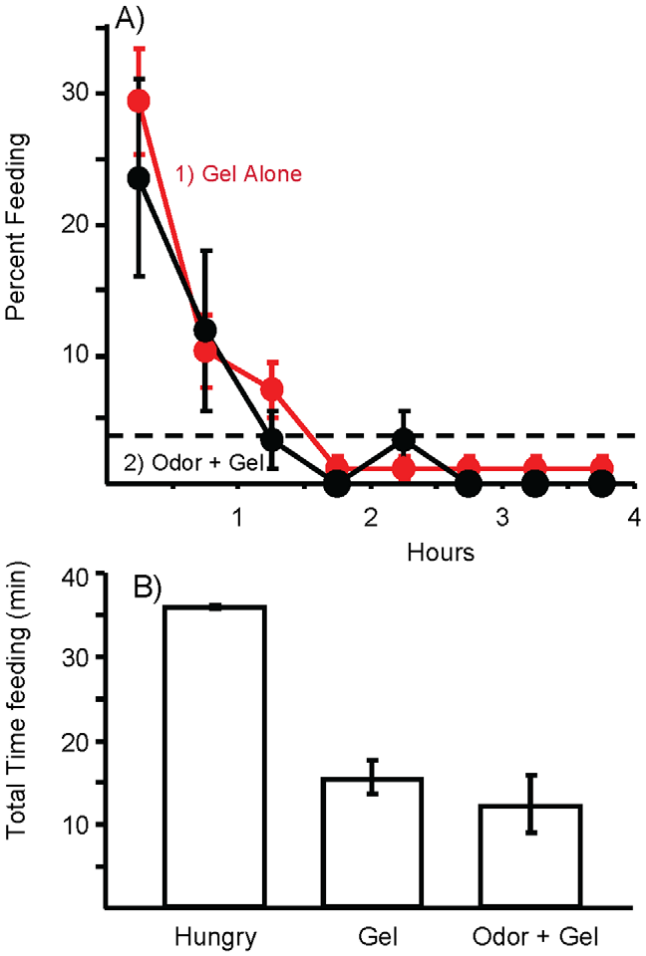
Injecting non-nutritive gel into the anterior gut inhibits feeding. A) 1: The effects of 2.5 g of gel alone (*N* = 28) or of 2: the gel plus a 2 hr pre-exposure to food odor (*N* = 10) were similar. In both conditions, feeding declined rapidly within the first 2 hr of the test, reaching steady-state levels (dashed line) by the end of the second hour. By contrast, hungry animals reached the steady-state only at the end of the 4 hr test (see Fig. 1). B) Comparison of the total time devoted to feeding during the 4 hr test in hungry animals, and in animals treated with either gel alone, or with gel plus food odor. The gel treatment significantly reduced the total time spent feeding (*p*<0.001, *t*(86) = 7.29). There was no difference in the total time spent feeding when animals were pre-exposed to food odor and also treated with the gel (*p* = 0.42, *t*(36) = 0.82; two-tailed *t*-tests with Bonferroni corrections). Standard errors are shown.

Previous work showed that when hungry *Aplysia* of the size used in these experiments have access to food, they eat a mean of 2.5 g of seaweed during the meal that initiates the steady-state inhibition [27]. To determine whether mechanical stimuli in the anterior gut sensing the food eaten contribute or account for entry into the steady-state, 2.5 g of a non-nutritive gel was injected into the anterior gut 10 min before a 4 hr access to food. This treatment produced a significant decrease in the time spent feeding (Fig. 3). Nonetheless, the treatment could not alone induce the steady-state inhibition, since animals responded well to the food at the start of the meal. During the first 2 hr of the test, feeding gradually decreased, and animals reached steady-state levels of feeding by the start of the third hour. Thus, bulk stimuli similar to those during a meal contribute to entry into the steady-state, but cannot alone account for entry into the steady-state.

Could the combination of bulk and food odor together account for entry into the steady-state? To examine this possibility, animals were pre-exposed to the odor of food for 2 hr, as well as being injected with 2.5 g of non-nutritive gel into the anterior gut 10 min before access to food. Feeding was then examined for 4 hr. There were no significant differences between animals treated with the gel alone, and those treated with the gel plus food odor, indicating the combined effect of the two factors inhibiting feeding is no better than is the effect of the gel alone (Fig. 3).

### L-arginine

The data above suggest that in addition to food odor and bulk in the gut, post-ingestive nutritive stimuli could contribute to establishing steady-state inhibition. A previous report [34] showed that the amino acid L-arginine can act as a post-ingestion stimulus inhibiting feeding. At physiological concentrations (a 10 µM increase in the extracellular fluid), L-arginine alone did not inhibit feeding in hungry animals. However, in animals that were already in the steady-state, a physiological increase in L-arginine concentration interacted with other inhibitory stimuli and amplified their inhibitory effect. In hungry animals, L-arginine was able to inhibit feeding and speed the entry into the steady-state only when the hemolymph concentration of L-arginine was increased by 2.5 mM, more than two orders of magnitude greater than the physiological increase caused by a meal [34].

**Figure 4:**
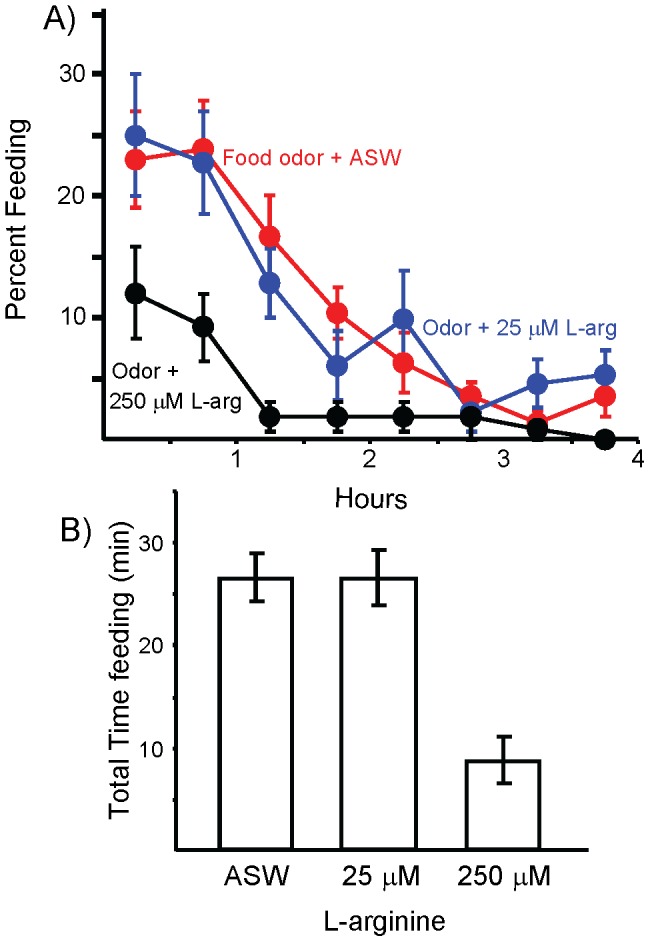
L-arginine augments inhibition caused by the odor of food. In all experiments, animals were food-deprived for 3 days, and were then allowed *ad-libitum* access to food for 4 hours. Animals were exposed to food odor for the two hours before the animals were permitted to eat food. In addition, animals were injected either with ASW (*N* = 37), or with solutions that raised the hemolymph L-arginine by 250 µM (*N* = 18) or 25 µM (*N* = 22). Data on the effects of food odor after ASW treatment replicate the data in Figs. 1A and 2. Standard errors are shown. A) The mean percent time spent eating during each half hour of the 4 hours. B) The total time spent feeding during the 4 hours. There were significant differences in the total time spent feeding between the three groups (*p*<0.001, *F*(2,74) = 13.2; one-way analysis of variance). A post-hoc test (Student-Newman-Kuels) showed that at α = 0.05, animals treated with 250 µM L-arginine ate significantly less than did animals in the other two groups, with no significant difference between animals treated with ASW or with 25 µM L-arginine. Nonetheless, the data in A suggest that 25 µM L-arginine might have had an inhibitory effect during the second hour of the meal. We therefore tested the effect of 25 µM L-arginine against the effect of ASW for each of the four hours of the meal. This comparison used only animals treated with ASW that were run on the days that the effect of 25 µM L-arginine was examined. Pre-treatment with 25 µM L-arginine indeed augmented the effect of pre-exposure to odor during the second period of four hours of the experiment (*p* = 0.04, *t*(40) = 2.09), without affecting feeding during the first (*p* = 0.95, *t*(40) = 0.51, third (*p* = 0.85, *t*(40) = 0.15) or fourth (*p* = 0.08, *t*(40) = 1.77) hours.

We examined the possibility that lower concentrations of L-arginine, which were not alone effective in regulating feeding [34], could also affect entry into the steady-state when L-arginine is present along with other effective stimuli. L-arginine could amplify the inhibitory effect of food odor, and thereby contribute to entry into the steady-state. Hungry animals were exposed to food odor for two hours before allowing the animal access to food for 4 hrs. Animals were also injected with L-arginine to cause an increase of either 250 µM or 25 µM in the hemolymph. Controls were treated with ASW. The pre-exposure to food odor produced a modest decrease in feeding when animals were subsequently allowed to eat, with respect to feeding in controls (compare Figure 4, food odor + ASW, with hungry animals in Fig. 1), confirming that food odor inhibits feeding in hungry animals. Treatment with 250 µM L-arginine significantly inhibited feeding (Fig. 4). Animals treated with 250 µM L-arginine and exposed to food odor ate an abbreviated meal, and spent significantly less time eating during both the first and second hours than did animals exposed to the food odor alone. However, since animals treated with food odor plus L-arginine did eat more than do animals in the steady-state when first given access to food, these experiments also indicate that additional stimuli must further contribute to entry into the steady-state.

By contrast to the effects of 250 µM L-arginine, the effects of 25 µM L-arginine, which is close to the increase caused by feeding [34], were modest. There was no significant different in the total time spent feeding, and feeding during the first, third and fourth hours of feeding were similar. However, comparing feeding during the second hour after 25 µM L-arginine to that after ASW treatment showed a significant reduction after L-arginine treatment, indicating that an increase in L-arginine approaching that seen after a meal could amplify the inhibitory effect of food odor. The lack of an effect during the first hour of food access suggests that additional stimuli arising from feeding during the first hour of feeding combined with the food odor and L-arginine, and the combination of these stimuli inhibited feeding.

**Figure 5:**
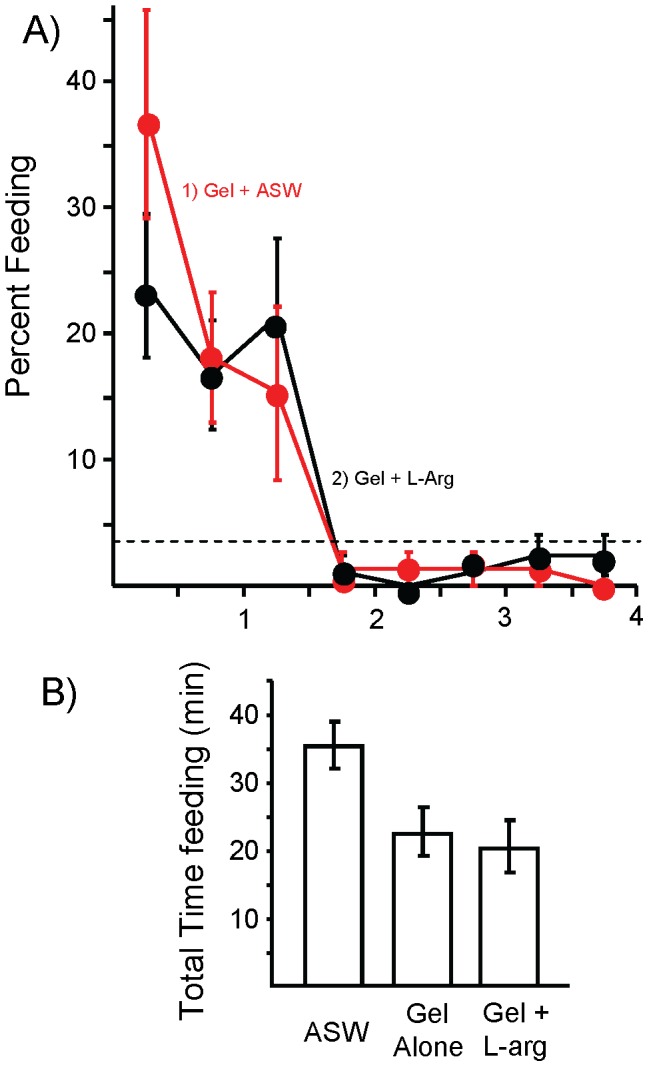
L-arginine does not amplify the inhibitory effect of gut fill. In this experiment, 3 groups of animals were examined. One (*N* = 8) was injected with ASW 10 min before a 4 hr feeding. A second (*N* = 12) was treated with 2.5 ml of gel injected into the crop before the 4 hr feeding. A third (*N* = 14) was treated with gel, plus an injection that raised the hemolymph concentration of L-arginine to 250 µM. Standard errors are shown. A) Percent time spent feeding in half-hour intervals over the 4 hr test. Data on animals treated with ASW are not shown. B) The total time spent feeding over the 4 hr test in the three groups. There was a significant difference between the three groups (*p*(2,31) = 0.036; *F* = 3.69; one-way analysis of variance). A post-hoc test (Student-Newman-Kuels) showed that at α = 0.05, animals treated with ASW ate significantly more than did animals in the other two groups, with no significant difference between animals treated with gel alone, or with gel and L-arginine.

We also examined whether treatment with 250 µM L-arginine could amplify the inhibitory effect of gut fill. In an experiment examining the effects of gut fill alone, versus gut fill plus pre-treatment with 250 µM L-arginine, there was no significant difference between the two groups, indicating the combined effect of the two factors inhibiting feeding is no better than the effect of the gel alone (Fig. 5). Thus, L-arginine can amplify the effect of food odor, but not of gut fill.

### Glucose

**Figure 6:**
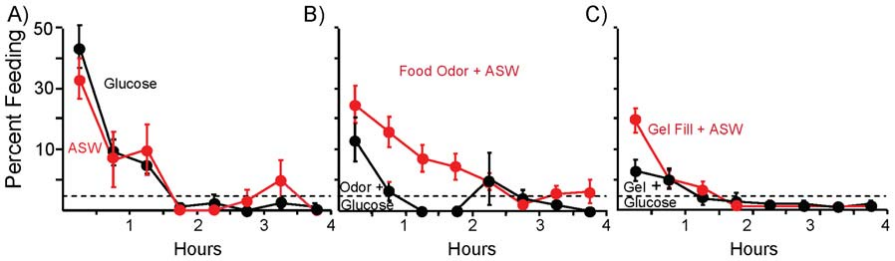
Glucose amplifies the inhibitory effects of food odor and of gel fill. Animals were treated with glucose 1 hr before testing feeding**.** *A*) There was no significant difference between the effects of glucose or of ASW on entry into the steady-state. *B*) Glucose amplified the inhibitory effects of food odor. During the first 2 hours of the meal, animals treated with glucose ate significantly less than did controls (*p* = 0.05, *t*(27) = 2.02). There was no significant difference in the subsequent two hours (*p* = 0.60, *t*(27) = 0.54). *C*) Glucose treatment also amplified the inhibitory effect of gut fill at the start of the meal. During the first half hour, glucose-treated animals ate significantly less (*p* = 0.007, *t*(44) = 2.79 than did controls, with no significant difference (*p* = 0.99, *t*(44) = 0.008); all tests are two-tailed *t*-tests) in the subsequent period. The dashed line shows steady-state feeding. Standard errors are shown.

We also examined whether a second metabolite, glucose, could contribute to establishing steady-state inhibition. Previous work [20] had shown that glucose levels in *Aplysia* hemolymph are physiologically regulated at 2–3 mg/dl, and that glucose levels in the hemolymph are increased after eating some seaweeds. However, an approximate doubling of the glucose level (addition of 320 mg/kg) does not affect feeding behavior [20]. We confirmed this finding. As in the previous study [20], animals were injected with 320 mg/kg glucose. Hungry animals were treated with glucose 1 hr before testing feeding with a 4 hours access to food. Controls were injected with ASW. There was no significant difference in the time spent feeding between animals treated with glucose or with ASW, during either the first or the second 2 hr of the 4 hr access to food (Fig. 6A). Values in both were comparable to those in untreated hungry animals (see Fig. 1). In both groups the percent time feeding declined over the 4 hr test, with values comparable to those in untreated hungry animals. Thus, glucose alone had no effect on feeding.

Could glucose amplify the effects of other stimuli that affect feeding as does L-arginine? To test this possibility, glucose-treated or ASW-treated animals were also exposed to food odor for the two hours preceding access to food. Glucose amplified the inhibitory effect of food odor (Fig. 6B). Glucose treatment of animals in the presence of food odor produced a more rapid decrease in feeding than did the food odor alone. Glucose-treated animals reached the steady-state within the first hour of access to food. Animals that had been treated with glucose devoted significantly less time to feeding during the first 2 hours of food access, with no significant difference during the second two hours, when both groups had entered the steady-state.

Glucose treatment differed from treatment with L-arginine, in that it also amplified the effect of non-nutritive gel in the gut (Fig. 6C). Because the inhibitory effect of gel alone is much larger than is the effect of food odor, the additional effect of glucose was seen only at the start of the meal, before the gel fill alone had caused the animals to enter the steady state. Glucose caused a significant decrease in the time spent feeding during the first 0.5 hr of food access, with no subsequent differences between glucose treated or control animals.

The amplification by glucose of the effects of gut fill or of food odor could not completely account for the entry into the steady-state, since there was still a reduction in feeding to the steady-state at the start of the meal. Thus, additional stimuli provided by food at the start of the meal contribute to entry into the steady-state. We examined the possibility that the combination of glucose, food odor and gut fill could completely account for entry into the steady-state. However, there was no significant difference between the effects of glucose plus gut fill and the effect of the addition of food odor to these two stimuli (not shown).

### Exit from the Steady-state

#### Effect of food odor

A previous report [27] showed that steady-state inhibition is maintained for as long as food is available. Removal of the food leads to a gradual decline of the steady-state over 24 hours, at the end of which animals respond to the re-introduction of food as do hungry animals that have been food deprived for a number of days. It was also shown that the presence of food odor in the water for 24 hr maintains the steady-state, so that when food is made accessible again animals respond as though the food had always remained accessible [27]. The effect of food odor in maintaining the steady-state was not examine d for periods longer than 24 hrs. We therefore examined how long food odor alone can effectively maintain the steady-state.

First, we replicated the previous finding that 24 hr of access to food odor maintains the steady-state. We then examined whether 48 hr or 72 hr of exposure to food odor are effective in maintaining the steady-state.

As in previous experiments [27, 34], animals were maintained with *ad libitum* food access for 72 hrs. The food was then removed, and placed behind a partition preventing animals from touching the food, but allowing the animals to smell the food. Food was then restored after 24, 48 or 72 hrs, and feeding was examined for 4 hrs.

**Figure 7:**
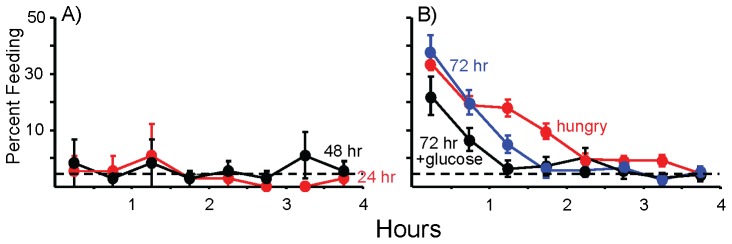
Identification of variables controlling the maintenance of the steady-state inhibition. A) Animals that had steady-state food access were prevented from eating, by placing the food behind a net partition, thereby allowing animals to smell the food, but not to touch or eat it. Animals were then permitted to eat for 4 hr either 24 or 48 hr later. The graph shows the percent time feeding during the 4 hr of access to food. Exposure to the food odor alone for either 24 hr or 48 hr preserved the steady-state inhibition. For both 24 hr and 48 hr exposures to food odor, there were no significant differences in the total time spent feeding during the first versus the fourth hour of access to the food (for 24 hr: *p* = 0.3; *t*(5) = 1.0; for 48 hr: *p* = 0.58, *t*(5) = 0.60; two-tailed paired *t*-test). Thus, food odor alone preserves the steady-state for 48 hr. *B*) By contrast, after a 72 hr exposure to food odor, animals ate vigorously when given access to food. There was a significant difference between the percent time feeding during the first and 4^th^ hour of access to food (*p*<0.001; *t*(19) = 9.78; two-tailed paired *t*-test), as would be expected if access to food initiated a meal. Thus, food odor does not preserve the steady-state for 72 hr. The graph shows the percent feeding in animals for half-hour intervals during 4 hr of food access, and compares data from animals exposed to 72 hr of food odor with data from hungry animals (data are identical to those in Fig. 1, and are shown again for comparison). The graph shows that after 3 days of exposure to food odor, there is a more rapid entry into the steady-state than in hungry animals. For the second hour of access to food, animals that had been exposed to food odor ate significantly less than did the hungry controls (*p*<0.001, *t*(79) = 5.75, two-tailed *t*-test). Thus, the 72 hr exposure to food odor still has an inhibitory effect on feeding. The graph also shows the effect of injecting animals with glucose before providing access to food. There was a significant decrease in feeding over the 4 hr feeding test, as shown by a significant decrease in feeding from the first to the fourth hour of the test (*p*<0.001; *t*(15) = 4.56; two-tailed paired *t*-test). The glucose treatment caused a significant (*p* = 0.005, *t*(34) = 2.97; two-tailed *t*-test) decrease in feeding during the first 2 hr of the test session, with respect to animals treated only with 72 hr exposure to food odor. The dashed line shows steady-state feeding. Standard errors are shown.

The steady-state inhibition was maintained by both 24 hr and 48 hr of exposure to food odor. During the 4 hr test of feeding, there were no significant decreases in feeding from the start to the end of the test. Throughout, animals responded as though the steady-state inhibition had been maintained. Thus, we have confirmed the previous observation that food odor alone maintains the steady state for 24 hr, and we have extended this observation to a 48 hr exposure to food odor (Fig. 7A).

By contrast to the maintenance of the steady-state after 48 hr of exposure to food odor, 72 hr of exposure to food odor did not maintain the steady-state inhibition of feeding (Fig. 7B). Thus, reintroducing food after 72 hrs of exposure to food odor caused a significant increase in feeding during the first hr of food access, as shown by a comparison of feeding in the first and fourth hour. However, the presence of food odor still had a partial inhibitory effect on feeding, as shown by a more rapid decrease in feeding in animals exposed to food odor, with respect to hungry controls (Fig. 7B). This was shown by a significant decrease in feeding during the second hour of the test in animals exposed to 72 hr food odor, with respect to hungry controls.

#### Glucose amplifies the inhibitory effect of food odor

The data above show that food odor alone can maintain the steady-state inhibition for up to 48 hr. Since in the presence of food the steady state is maintained indefinitely, additional stimuli must also signal the reduction of feeding in the steady state. When *Aplysia* first enter the steady-state, increases in hemolymph L-arginine and glucose concentrations can amplify the effects of other stimuli (see Figs. 4, 6). A previous report [34] showed that a 10 µM increase in L-arginine can amplify the inhibitory effect of food in the environment in the maintenance of the steady-state inhibition, and thereby contribute to the maintenance of the steady-state beyond 48 hrs. We tested the possibility that glucose may also be a post-ingestion stimulus that could amplify the inhibitory effect of food in the environment.

As in the previous experiment, animals that had been in the steady state were placed in an environment in which food was inaccessible, but was present behind a partition for 72 hr, thereby providing food odor. Animals were injected with glucose to obtain a concentration of 320 mg/kg above that in untreated controls 2 hr before restoring food and measuring feeding for 4 hr. Glucose treatment significantly reduced feeding during the first 2 hr of access to food, indicating that glucose could contribute to the maintenance of the steady-state, along with food odor. However, the combination of glucose plus food odor was not sufficient to maintain the steady-state, as shown by a significant reduction of feeding from the first to the fourth hour of the test (Fig. 7B). Thus, additional factors provided by the continuous access to food must contribute to the indefinite maintenance of the steady-state.

#### L-arginine and glucose alone affect maintenance of the steady-state

**Figure 8:**
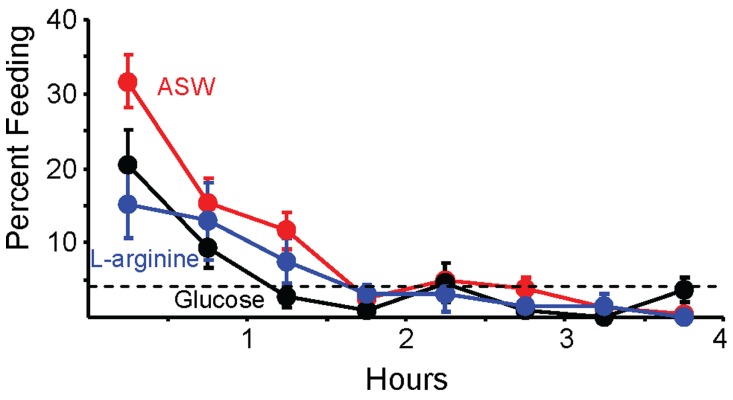
The effects of L-arginine (250 µM) or of glucose (320 mM) alone on the preservation of the steady-state. Animals were treated with ASW, glucose or L-arginine 5 hours after removing food, and were tested 24 hrs after the food was removed. The percent time spent feeding for each half hour of the 4 hr test is shown. Standard errors are shown. In all treatments, there were significant differences between the first and last half-hour of the 4 hr test session (for ASW: *p*<0.001, *t(*39) = 8.71; for L-arginine: *p* = 0.003, *t*(21) = 3.36; for glucose: *p* = 0.003, *t*(17) = 3.350; two-tailed paired *t*-tests), indicating that the steady-state was not preserved. Nonetheless, both L-arginine and glucose caused significant decreases in feeding during the first 2 hrs of the test session. A one-way analysis of variance showed significant differences in the total time spent eating during the first two hours between the three treatments (*p* = 0.028; *F*(2,77) = 3.74). A post-hoc test (Student-Newman-Kuels) showed that at α = 0.05, animals treated with ASW ate significantly more during the first two hours than did animals in the other two groups, with no significant difference between animals treated with L-arginine and with glucose.

Both glucose (see above) and L-arginine [34] are able to amplify the inhibitory effect of food odor, so that after food odor alone is no longer effective in absolutely maintaining the steady-state, either glucose or L-arginine can somewhat amplify its effect. Could either glucose or L-arginine act as does food odor, and themselves alone maintain the steady-state? To test these possibilities, food was removed from animals that had had continuous access to food for the previous three days. Five hours after removal of the food, animals were injected with either ASW or with 320 mg/kg glucose, or with 250 µM L-arginine. Food was restored 19 hrs later (24 hrs after removal of the food), and the time spent feeding was measured over the next 4 hrs (Fig. 8). Glucose and L-arginine differed from food odor, in that neither glucose nor L-arginine preserved the steady-state. In the presence of glucose or L-arginine, there were decreases in feeding over the 4 hr experiment. Nonetheless, the treatments with glucose or with L-arginine caused significant decreases in feeding during the first 2 hrs of food access, with no significant differences in the second 2 hrs. Thus, the effects of these two nutritional factors were present, but were much smaller than was the effect of food odor.

## Discussion

It was previously thought that *Aplysia* eat large meals [1, 11], as do mammalian grazers (*e.g.*, [23–25]). The animal’s anatomy is consistent with this hypothesis: *Aplysia* have a large crop that can store food equivalent to 25–30% of a hungry animal’s volume [12]. After the crop is filled, approximately 50% of the material is emptied within 24 hours, indicating that *Aplysia* can process large quantities of food [12]. Earlier experiments [1, 7, 12, 13], and observations of freely-behaving animals in laboratory aquaria and in the field [21, 22], were consistent with *Aplysia* eating large meals that are controlled by the inhibitory effects of crop fill, which counters the excitatory effects of food stimuli initiating feeding [7, 14]. Crop fill apparently causes release of neuropeptides [3], which inhibit feeding.

More recent work has found that *Aplysia* feeding is considerably more complex than previously thought. When isolated *Aplysia* have continuous access to food, they eat relatively little. During the steady-state inhibition, feeding is relatively inhibited and animals irregularly eat small meals which *in toto* fill 1–4% of the total time budget, eating a mean of 0.8 g per 6 hours [27]. The steady-state is maintained indefinitely, for as long as food remains available. Removal of the food causes a gradual decay of the steady-state over 24 hrs. Before the steady-state has completely decayed, animals are in an intermediate state, in which they have partially left the steady-state [27].

The two modes of *Aplysia* feeding can be explained as adaptations to different environments. As in other animals [35], *Aplysia* seem to be sensitive to whether food is constantly or patchily available. *Aplysia* are often found in abundance amid their food, seaweed. There is no need to find food, since it is always present [36]. However, they are also found when food is patchily present. They may then be buried in sand, or under rocks, some distance away from food [36]. Weather conditions can also strongly affect the availability of seaweed, which can wax and wane [36]). The large meals signaled by the sudden appearance of food seem to be designed as an adaptation to conditions when food is intermittently present. In these conditions, animals maximally fill the gut, and rapidly process food, building reserves for the future. By contrast, when food is abundant, animals can eat more moderately, eating only as much as needed to fulfill their modest metabolic needs.

### Many Stimuli Interact to Control Feeding

Early studies indicated that *Aplysia* feeding is controlled by only a few stimuli. Food elicits an arousal state, and also elicits feeding behavior [1]). Receptors responding to food may undergo sensory adaptation [7], thereby reducing responsiveness. Food in the gut elicits excitation of feeding via activation of gut chemo-afferents [7], followed by a larger inhibition caused by the activation of mechano-afferents [11–13]. Feeding was thought to be controlled by the interaction between excitatory and inhibitory stimuli [10]. Control of feeding by food and gut fill leaves little room for metabolic stimuli, such as L-arginine or glucose, to modulate the behavior. However, the identification of a second mode of feeding opens up the possibility for regulation of feeding by additional stimuli, some of which have been identified in the present manuscript. When food has recently been removed, and animals have partially left the steady-state [27], feeding may be regulated by a combination of stimuli that govern the steady-state, plus gut fill and sensitivity to food.

In addition to stimuli governing feeding in different conditions of food availability, the presence of pheromones secreted by potential mates also strongly modulate feeding [28]. When mature *Aplysia* are in contact when one another, the percent time feeding is increased from 1–4% of the time, to over 20% of the time. The increased feeding may be an adaptation for egg-laying. Mature, fertile *Aplysia* lay egg-masses that weigh a mean of 5% of the animals’ weight every few days [37].

### Sites of Action of Stimuli Affecting *Aplysia* Feeding

This and previous studies have identified a number of stimuli that affect *Aplysia* feeding. The sites of action at which they affect feeding are partially known.

#### Anterior gut stimuli

Mechanostimuli that arise from gut fill inhibit feeding. These stimuli contribute to the initiation of the steady-state (Fig. 3), and are the major inhibitory stimuli when animals eat large, intermittent meals [11, 12]. The anterior gut contains many peripheral neurons that send axons into the esophageal nerve travelling to the buccal ganglia [38, 39]. Some of these are presumably mechanoreceptors that respond to gut fill and that contribute to inhibition of feeding. Afferents containing *Aplysia* neuropetide Y (apNPY) are located on the esophageal nerves, and treatment with apNPY causes inhibition of feeding [3]. apNPY is thought to act in part on cerebral-buccal interneuron-2 (CBI-2), a command-like interneuron that is activated by food. The peptide causes CBI-2 to elicit egestive responses in place of ingestive responses [3].

The anterior gut also contains chemoreceptors that are excitatory and whose activation contributes to arousal of feeding [7]. The possible contribution of gut chemoreceptors to the initiation or maintenance of the steady-state inhibition has not been examined.

#### Food Chemostimuli

Food odor is sensed by the rhinophores [31], and has both excitatory and inhibitory effects on feeding. Pheromones that excite feeding are also sensed by the rhinophores [40]. In hungry animals, food odor induces appetitive (food-finding) feeding behaviors [32, 41], as well as arousal of feeding [2]. Food odor induces firing of neurons C-PR and MCC [15, 42], whose effects on follower neurons are partially responsible for appetitive behaviors and aspects of food arousal [2, 15, 43]. Food taste is sensed by chemoreceptors on the lips, and the combined activation of these chemoreceptors and of lip mechanoreceptors induces a consummatory feeding response, biting [44]. Tasting food also induces C-PR and MCC firing [2], along with activity of command-like neurons that synapse on central pattern generator (CPG) elements that organize consummatory responses [45].

A prolonged exposure to food odor causes inhibition of feeding in place of excitation, in both hungry animals (Fig. 1), as well as in animals that are already in the steady-state (Fig. 7). When the rhinophores are ablated, the steady-state can be maintained by food that stimulates the lip and is then consumed [27], suggesting that maintained stimulation of lip afferents can inhibit feeding about as well as can rhinophore afferents. However, since these animals were able to consume food, it is also possible that post-ingestive stimuli contribute to the maintenance of the steady-state inhibition when the rhinophores are ablated. Combining food odor with lip stimulation did not cause a greater inhibition of feeding than did food odor alone (Fig. 1), suggesting that lip stimulation may act in place of food odor, but does not enhance its effect. The maintained inhibition of feeding by food odor alone for up to 48 hrs (Fig. 7) indicates that receptors to food odor do not desensitize over this period of time.

The long-lasting inhibitory effects of rhinophores and lip stimulation contrast with the short-lasting effects of these stimuli on mating. In hungry animals, a 4 hr exposure to food odor inhibits mating, presumably because the food odor induces food-finding behavior which conflicts with mating [28]. However, a 24 hr exposure to food odor no longer inhibits mating [28]. By contrast, adding food taste (touching the lips every 5 min for the 4 hrs preceding the test) to a prolonged food odor does inhibit mating [28]. Since our present experiments showed that receptors to food odor do not desensitize or adapt for at least 48 hrs (see Fig. 7), the lack of inhibition of mating by a 24 hr exposure to food odor could not be attributed to desensitization or sensory adaptation. The lack of effect of maintained food odor on mating is likely to result from central habituation of neurons activated by food odor in the neural pathway controlling mating, with a lack of habituation in the central pathway that accounts for inhibition of feeding. Central habituation of pathways responding to food touching the lips was previously shown [7] by comparing the MCC neuron activity in response to ipsilateral and contralateral touch of food, and showing a generalization of response decrement in both. Central habituation of excitatory effects of odor and smell, with a lack of habituation of inhibitory effects, could explain how the short-term excitatory effects of these stimuli are followed by a long-term inhibitory effect. This could be tested by directly recording from neurons receiving input from food that cause feeding arousal and that inhibit feeding. One would expect to observe habituation in the former, but not in the latter.

The neural sites at which food stimuli act to inhibit feeding are likely to differ from the sites at which these stimuli excite feeding. There are no data on the localization of neural mechanisms by which food odor and taste inhibit feeding.

#### Nutritional stimuli

Previous data [27] showed that hemolymph extracted from animals in the steady-state inhibits MCC activity, suggesting that post-ingestion increases in nutrients in the hemolymph may have a role in maintaining the steady-state. In the present work, the effects of 2 nutrients, L-arginine and glucose, were examined. In hungry animals, concentrations of glucose of approximately double that found in the hemolymph did not inhibit feeding [20, and Fig. 5A], and concentrations of L-arginine that are approximately 3 orders of magnitude above those found in hemolymph are needed to affect feeding [34]. However, both glucose and L-arginine have inhibitory effects on feeding in hungry animals when combined with the inhibitory effects of other stimuli, such as food odor (Figs. 4, 6) or gut fill (Fig. 6). In addition, either stimulus can act to partially maintain steady-state inhibition (Fig. 8), or to amplify the inhibitory effect of food odor, so that feeding is partially inhibited even when the effect of food odor wears off (Fig. 7B). It is likely that additional nutrients also act along with L-arginine and glucose in inhibiting feeding.

L-arginine is the precursor for the production of nitric oxide (NO) by nitric oxide Synthase (NOS) [46]. NO has been shown to inhibit two key neurons controlling different aspects of feeding behavior. C-PR has been described as a command neuron controlling food arousal [15], and B31/B32 are neurons that have a major role in deciding to initiate a consummatory feeding response, and in effecting the first stage of such a response [47]. Both C-PR and B31/B32 produce NO at rest, and NO production inhibits the cells [48]. The self-inhibitory effects of NO are revealed when the neurons are treated with blockers of NO action, which cause depolarization [48]. L-arginine is likely to modulate feeding by modulating NOS activity in C-PR and B31/B32, and perhaps also in additional neurons. Because C-PR is presynaptic to the MCC [49], the inhibition of the MCC by hemolymph from animals in the steady state that was previously found [27] may be partially the result of L-arginine regulation of C-PR.

### Is *Aplysia* Feeding Still a Useful Simple Model System?

*Aplysia* was chosen as a model preparation for studying the neural basis of motivation, because it showed all of the features of motivated behaviors in higher animals, in a simpler form, in an animal whose nervous system is accessible to a detailed cellular analysis [1]. The finding that initiation and termination of feeding are controlled by a small number of stimuli [6, 14, 7, 13] strengthened the notion that *Aplysia* feeing is an excellent model system for examining motivation. Over the years, the cellular basis of many characteristics of motivational control have been identified (e.g., [2, 3, 16]), confirming the utility of *Aplysia* feeding as a model system.

The findings that *Aplysia* feeding is controlled by many stimuli that have complex interactions, and which may operate in different environmental conditions, indicates that *Aplysia* feeding is not a simple system. Why continue studying control of *Aplysia* if it may not be much less complex than is control of feeding in higher animals? The access to cellular mechanisms underlying control of feeding still makes *Aplysia* an attractive experimental preparation. Cellular properties of neurons in hypothalamic and brainstem nuclei controlling mammalian feeding have also been investigated (e.g., [50–52]). However, these neurons are many synapses upstream from neurons that directly control behavior, and it is therefore difficult to understand how the regulation of these neurons leads to changes in behavior. In contrast, neurons in *Aplysia* that respond to feeding-related signals are synaptically close to, or are identical with, neurons that organize feeding behaviors, or that innervate muscles that affect feeding. Thus, the connection between cellular mechanisms controlling these neurons and the control of feeding behavior is more easily understood (e.g., [3, 34, 48]). Information from the *Aplysia* feeding control system is likely to be a harbinger of information on mammalian systems, which will be revealed as more becomes known about the complex circuits that connect mammalian hypothalamic and brainstem neurons that respond to feeding stimuli with neurons that directly produce or inhibit feeding behavior.
